# A Heart Image Segmentation Method Based on Position Attention Mechanism and Inverted Pyramid

**DOI:** 10.3390/s23239366

**Published:** 2023-11-23

**Authors:** Jinbin Luo, Qinghui Wang, Ruirui Zou, Ying Wang, Fenglin Liu, Haojie Zheng, Shaoyi Du, Chengzhi Yuan

**Affiliations:** 1 School of Physics and Mechanical and Electrical Engineering, Longyan University, Longyan 364012, China; fjlcljb@sina.com (J.L.); wqh0597@126.com (Q.W.); zray_zou@126.com (R.Z.); lyxyy_w@126.com (Y.W.); liufenglin45@126.com (F.L.); 2 School of Software Engineering, Xi’an Jiaotong University, Xi’an 710049, China; 18792937600@139.com; 3 Institute of Artificial Intelligence and Robotics, Xi’an Jiaotong University, Xi’an 710049, China; dushaoyi@xjtu.edu.cn; 4 Department of Mechanical, Industrial and Systems Engineering, University of Rhode Island, Kingston, RI 02881, USA

**Keywords:** medical imaging, segmentation, attention mechanism, inverted pyramid, contextual information

## Abstract

In the realm of modern medicine, medical imaging stands as an irreplaceable pillar for accurate diagnostics. The significance of precise segmentation in medical images cannot be overstated, especially considering the variability introduced by different practitioners. With the escalating volume of medical imaging data, the demand for automated and efficient segmentation methods has become imperative. This study introduces an innovative approach to heart image segmentation, embedding a multi-scale feature and attention mechanism within an inverted pyramid framework. Recognizing the intricacies of extracting contextual information from low-resolution medical images, our method adopts an inverted pyramid architecture. Through training with multi-scale images and integrating prediction outcomes, we enhance the network’s contextual understanding. Acknowledging the consistent patterns in the relative positions of organs, we introduce an attention module enriched with positional encoding information. This module empowers the network to capture essential positional cues, thereby elevating segmentation accuracy. Our research resides at the intersection of medical imaging and sensor technology, emphasizing the foundational role of sensors in medical image analysis. The integration of sensor-generated data showcases the symbiotic relationship between sensor technology and advanced machine learning techniques. Evaluation on two heart datasets substantiates the superior performance of our approach. Metrics such as the Dice coefficient, Jaccard coefficient, recall, and F-measure demonstrate the method’s efficacy compared to state-of-the-art techniques. In conclusion, our proposed heart image segmentation method addresses the challenges posed by diverse medical images, offering a promising solution for efficiently processing 2D/3D sensor data in contemporary medical imaging.

## 1. Introduction

In contemporary times, medical imaging technology has progressively evolved into an indispensable adjunct tool for physicians, aiding in disease examination, diagnosis, and treatment facilitation. Medical images are typically obtained through a range of medical imaging techniques, including X-rays, Magnetic Resonance Imaging (MRI), Computed Tomography (CT), and Ultrasound Diagnostic Techniques [1]. These images play a crucial role in enabling doctors to visualize the morphology of diverse tissues and organs within the human body, thereby facilitating a rapid comprehension of pathological changes occurring within the patient’s body. Medical images offer the means for physicians to assess organ conditions and identify lesions in the early stages of treatment, thereby enabling the formulation of appropriate treatment strategies. Additionally, throughout the subsequent treatment processes, physicians can monitor the specific impacts of the ongoing treatment plan by tracking alterations in the medical images. Moreover, medical images play a pivotal role in the establishment of pertinent medical databases, which document various cases and corresponding treatment modalities. These databases serve as references for analogous cases and contribute to the continuous enhancement of treatment plans, thereby propelling advancements within the realm of medicine.

Medical image segmentation encompasses the delineation of regions of interest, such as pathological tissues and distinct organs, within specific medical images. This process effectively accentuates the relevant features within the images. In various medical image analysis endeavors, such as image registration, labeling, and object tracking, the segmentation of medical images assumes paramount importance. This is because these tasks necessitate the segmentation of anatomical structures, subsequently enabling subsequent analyses tailored to diverse task requisites. As a result, medical image segmentation holds extensive practical utility and substantial research importance within the medical domain. It assumes a pivotal role in aiding diagnostic and treatment procedures by facilitating accurate identification and characterization of pertinent areas.

Presently, the majority of medical image segmentation is carried out manually by medical professionals using specialized software to delineate organ boundaries and annotate regions of interest. However, the intricate nature of human tissue structures, coupled with potential noise in medical imaging, can impede accurate annotations. Consequently, this approach places a substantial burden on clinicians, requiring significant time and effort for marking target areas within patients’ medical images. Moreover, due to the subjective nature of individual doctors’ judgments, achieving consistent segmentation results for the same image is challenging. In contrast, computer-based medical image segmentation offers a more objective alternative. Once a specific algorithm is selected and relevant parameters are determined, the segmentation outcomes remain consistent across the same medical image. Therefore, the investigation of medical image segmentation algorithms holds paramount importance in advancing the medical field. Not only does it relieve doctors of this labor-intensive task, but it also ensures the stability and reproducibility of segmentation results. However, medical segmentation encounters certain challenges. Firstly, the variability in organ sizes, disparities in category distributions, and diverse resolutions across medical images acquired from different imaging modalities demand increased contextual information to bolster segmentation precision. Secondly, given that medical images inherently possess more prior knowledge than natural images (such as consistent organ locations and spatial relationships), effectively harnessing this prior knowledge is a burgeoning research area. Addressing these challenges necessitates further exploration to elevate segmentation accuracy and refine segmentation outcomes.

The significance of accurate and efficient heart image segmentation lies at the core of advancing medical diagnostics and treatment planning. Precise delineation of the heart structures is essential for understanding heart anatomy, detecting abnormalities, and guiding interventions. However, despite the progress in medical imaging, heart image segmentation remains a challenging task due to several factors.

One prominent challenge is the intricate and dynamic nature of heart structures. The heart exhibits complex anatomical features, including chambers, valves, and vessels, which undergo deformations during the cardiac cycle. Traditional segmentation methods often struggle to adapt to these dynamic changes, leading to suboptimal results. Another critical consideration is the demand for segmentation accuracy in various clinical applications. From assessing heart function to diagnosing pathologies, the accuracy of segmentation directly influences the reliability of subsequent analyses. Existing methods may fall short in achieving the necessary precision, particularly in the presence of image artifacts, noise, or variations in patient anatomy. Moreover, the diverse modalities and imaging protocols used in heart imaging introduce additional complexities. MRI, CT, and echocardiography each present unique challenges, requiring adaptable segmentation techniques that can perform consistently across modalities.

While recent advancements in deep learning have shown promise in medical image segmentation, there is still a scarcity of specialized methods addressing the intricacies of heart image segmentation. Many existing approaches lack the specificity needed to handle the dynamic nature of heart structures and the nuances of heart imaging modalities. In this context, our work addresses these gaps by introducing a novel heart image segmentation approach that leverages multi-scale features and a position-aware attention mechanism. This methodology aims to enhance the precision and adaptability of heart image segmentation, contributing to more accurate and reliable analyses in clinical settings. Through our proposed method, we strive to advance the state of the art in heart image segmentation, ultimately improving patient care and clinical decision-making.

The motivation behind our efforts in heart image segmentation stems from the critical role it plays in advancing clinical diagnosis and medical applications. The challenges associated with heart image segmentation are multifaceted, primarily driven by the intricate nature of the heart’s anatomy and its dynamic behavior during the cardiac cycle.

(1) Anatomical complexity: The heart’s intricate structure, comprising chambers, valves, and myocardium, demands precise segmentation for accurate diagnosis. Conventional segmentation methods often struggle to capture the nuanced boundaries and interdependencies within the heart’s anatomy.
(2) Dynamic changes: The dynamic nature of the heart introduces additional challenges, as its morphology evolves throughout the cardiac cycle. Reliable segmentation must account for these variations to ensure accurate representation and analysis.
(3) Clinical significance: Accurate segmentation is pivotal for clinical applications, such as assessing heart function, detecting abnormalities, and guiding interventions. Suboptimal segmentation can lead to misdiagnosis and hinder effective treatment planning.

The proposed heart image segmentation method addresses these challenges by incorporating a multi-scale feature and attention mechanism within an inverted pyramid framework. This design choice allows our algorithm to capture contextual information across different resolutions, accommodating the intricacies of the heart’s anatomy and dynamic changes during the cardiac cycle.

Significance for clinical diagnosis lies in the following:

- Precision and efficiency: our method enhances the precision of heart image segmentation, providing clinicians with more accurate representations of the heart’s structures.
- Automation and workload reduction: automation of segmentation tasks reduces the manual workload for medical professionals, allowing for faster and more efficient image analysis.
- Treatment planning: accurate segmentation contributes to precise treatment planning, especially in interventions such as surgery or catheter-based procedures.

Significance for medical applications lies in the following:

- Research and education: Reliable segmentation facilitates research endeavors by providing detailed insights into heart morphology. It also serves as an educational tool for training medical professionals.
- Image-guided interventions: accurate segmentation is crucial for image-guided interventions, ensuring precise navigation and targeted treatment delivery.

In essence, our heart image segmentation method is not merely a technical advancement; it is a crucial enabler for improving the accuracy of cardiac diagnosis, enhancing treatment outcomes, and advancing medical research and education. Through its unique design tailored to the challenges of heart imaging, our method has the potential to significantly impact the field of cardiovascular healthcare.

Image segmentation entails identifying and isolating foreground regions of interest within an image, which exhibit distinct properties and are of human interest, thereby separating them from the background regions. This technique holds pivotal importance in the realm of computer vision and has garnered considerable attention since the 1970s. Throughout the evolution of image segmentation, solutions to this challenge can be broadly classified into two principal categories: traditional image segmentation methods and deep learning-based image segmentation approaches. Traditional methods [2] represent one category, while the other encompasses deep learning techniques [3].

(1) Image Segmentation based on Traditional Methods

Prior to the widespread adoption of deep learning techniques, a plethora of classical traditional segmentation methods had been developed by researchers. These methods can be broadly categorized into three types: region-based segmentation, boundary-based segmentation, and segmentation methods that integrate insights from other domains [2].

(I) Region-Based Segmentation Methods: This category involves segmenting images based on identifying similar characteristics within the image. One widely used technique within this category is thresholding. In thresholding, predefined threshold values are used to compare each pixel in the image, segmenting it based on the outcome of these comparisons. Thresholding is characterized by its simplicity, computational efficiency, and speed.
(II) Boundary-Based Segmentation Methods: These methods achieve segmentation by detecting boundaries within the image. Edges, representing abrupt changes in pixel values, are typically detected using differential operators.
(III) Segmentation Incorporating Knowledge from Other Fields: Given the absence of a universal segmentation theory in traditional image segmentation, methods that incorporate knowledge from other domains have emerged. This involves applying theories from diverse fields to the segmentation task. For instance, mathematical morphology-based boundary detection algorithms, fuzzy set theory-based methods, and wavelet transform-based boundary detection methods fall into this category.

In summary, traditional segmentation methods offer simplicity, speed, and effectiveness on specific images. They possess strong interpretability and are readily comprehensible. However, due to their design for specific image attributes, no single traditional method can accommodate all image types, particularly complex images and extensive datasets.

(2) Image Segmentation based on Convolutional Neural Networks (CNNs)

In recent years, the rapid advancement of deep learning has prompted an increasing number of researchers to harness its potential for computer vision tasks. Notably, in 1988, LeCun et al. introduced LeNet-5 [4], a network tailored for handwritten character recognition. LeNet-5 employs a sequence of convolutional and pooling operations to extract features from input images, subsequently employing fully connected layers for character classification. This pioneering work set the stage for deeper exploration in the field. In 2012, AlexNet achieved groundbreaking success in the ImageNet LSVRC-2010 competition [5]. While the network structure shared similarities with LeNet-5, it boasted a more intricate architecture, including additional layers and parameters. This led to a performance boost exceeding 10%, sparking considerable enthusiasm within the academic community. Building upon AlexNet’s success, subsequent researchers developed more sophisticated neural architectures, such as VGGNet [6] and ResNet [7], which serve as foundational frameworks for extracting semantic features in diverse computer vision tasks.

In the initial stages, image segmentation involved direct application of CNN structures to the segmentation task. As CNNs were initially designed for image classification, they yielded one-dimensional feature vectors as outputs. These vectors could either directly classify input images or be employed for segmenting images into individual pixels. To predict the class of a specific pixel, the network required input from a region centered around that pixel, wherein convolution, pooling, and normalization operations were applied. To predict the segmentation of an entire image, a sliding window sequentially covered different regions of the image, predicting the central pixel by sliding the window and repeating this process. However, this approach proved computationally inefficient and demanding in terms of storage space. Furthermore, the size of the sliding window directly impacted segmentation effectiveness, striking a balance between capturing relevant information and maintaining a feasible receptive field size.

Addressing these challenges, Long et al. [8] introduced the first end-to-end fully convolutional network (FCN), which capitalized on semantic information. The FCN architecture entails fine-tuning large classification networks such as VGGNet and ResNet. To ensure that output images directly represented 2D segmentation outcomes, FCN incorporated deconvolutional layers in lieu of fully connected layers. This innovation aligned output dimensions with segmentation needs. Mainstream image segmentation algorithms later adopted deconvolutional operations for their ability to retain image characteristics. Additionally, FCN pioneered the skip structure, which amalgamates low-level and high-level features via feature extraction layer fusion to enhance segmentation results.

Subsequent to the inception of FCN, researchers embarked on a trajectory of diverse enhancements building upon this fundamental framework. However, due to the utilization of deconvolutional layers for feature map resolution restoration within the FCN’s decoder, the resultant segmentation outcomes continue to exhibit a deficiency in capturing intricate details. In this context, SegNet [9] emerged as an innovative advancement to the FCN paradigm, meticulously recording the indices corresponding to each layer of max-pooling during the encoding phase of the network. This innovative approach empowers the network to execute anti-pooling operations contingent upon the corresponding indices during the decoding process, culminating in segmentation outcomes characterized by enhanced smoothness and intricate delineation. Furthermore, UNet stands as another archetypal framework in the domain of medical image segmentation [10]. This model employs a symmetrical encoder–decoder configuration, concomitant with the utilization of skip connections, to seamlessly concatenate features of identical resolution originating from both the encoder and decoder pathways. This mechanism facilitates UNet in the retrieval of both low-level and high-level semantic information, which might be otherwise compromised during the encoding phase. Consequently, it engenders more precise segmentation outcomes, while simultaneously curtailing the requirement for an extensive corpus of training images.

Given that CNN employes convolutional kernels to encompass the receptive field in an image, the crucial requirement of encompassing features of diverse sizes within an image becomes evident [11,12,13]. In response to this challenge, Zhao et al. [14] pioneered the PSPNet architecture, highlighting the paramount significance of the pyramid pooling module (PPM) designed to capture multi-scale contextual information. Subsequent to feature extraction, the PPM performs pooling operations on features with distinct grid sizes to capture context information across varying scales. The Deeplab series, evolving from this premise, presents an array of network architectures meticulously crafted to amass enhanced contextual information [15]. Central to this series is the pivotal Atrous Spatial Pyramid Pooling (ASPP) module, which traces its origins to Deeplab v1. In the pursuit of mitigating semantic information loss attributed to downsampling in CNNs, Deeplab v1 introduced dilated convolutions as a means to augment the receptive field. Nevertheless, the scope of enhancement in Deeplab v1 was limited to a solitary application of dilated convolution for receptive field augmentation, whereas Deeplab v2 introduced the innovative ASPP mechanism. ASPP strategically employs dilated convolutions featuring distinct dilation rates on a shared input feature, orchestrating the capture of context information spanning multiple scales. These features embodying multi-scale characteristics are subsequently amalgamated within distinct branches, undergoing convolutional operations followed by concatenation to yield the ultimate outcome. Lin et al. introduced the Feature Pyramid Network (FPN) architecture [16], which is adept at acquiring features of varying levels from the pyramid-like structure and orchestrating their fusion through lateral connections spanning top to bottom. Subsequently, the amalgamated features are transmitted to the subsequent level. Moreover, FPN extends its functionality to include predictions for each hierarchical level. This multi-tiered feature fusion mechanism assumes prominence in subsequent image segmentation endeavors. Illustratively, Kirillov et al. [17] integrated Mask R-CNN [18] with FPN to address real-world segmentation challenges. Additionally, Zhou et al. [19] innovatively devised a 3D dilated convolution feature pyramid grounded in the FPN framework, orchestrating the amalgamation of context information to facilitate the recognition of objects of diverse dimensions.

The attention mechanism finds frequent application in computer vision to amplify model performance, drawing inspiration from human visual behavior, which naturally prioritizes significant regions within an image while disregarding background details. In the context of computer vision, this mechanism assigns heightened weight to potentially intriguing areas within an image, with greater significance accorded to vital regions, while de-emphasizing less informative portions. A prime example of this efficacy in image segmentation is showcased by the Squeeze-and-Excitation Network (SENet) [20]. In conventional convolutional networks, all channels within a feature map are treated as equal in importance. Yet, in practice, distinct channels carry varying degrees of significance. SENet resolves this concern by undertaking global feature map pooling, subsequently allocating distinct weights to individual channels. This practice steers the network’s attention towards the more pivotal segments of the input image.

Advancing this concept, the Convolutional Block Attention Module (CBAM) [21] introduces spatial attention encoding through extensive kernel convolution, effectively unifying spatial attention with channel attention. Another innovation, Coordinate Attention (CA) [22], decomposes channel attention into two one-dimensional feature encodings, aggregating features along disparate directions. Throughout this procedure, CA captures extensive interdependencies along one axis, while simultaneously conserving positional information along the orthogonal axis. This design endows the feature map not only with directional perception but also positional sensitivity. Fu et al. [23] present a method to independently learn context dependencies and pixel associations via self-attention mechanisms, operating across spatial and feature dimensions. Moreover, the inherent correlations present in distinct parts of the input are capitalized upon by integrating self-attention within the attention mechanism, resulting in enhanced performance through the learning of these correlations. Ye et al. [24] introduce the CMSA structure, wherein self-attention mechanisms guide the selection of 3D features to inform 2D image segmentation. Further elevating lung image segmentation outcomes, Kim et al. [25] fuse spatial and channel features through self-attention mechanisms atop the UNet architecture. This concept of self-attention also finds direct implementation in Transformer architecture.

(3) Image Segmentation based on Transformers

Owing to the intrinsic limitations of traditional convolutional neural networks in capturing extensive relationships within images, particularly for subjects characterized by substantial variations in texture, shape, and size, conventional CNN-based methods often encounter challenges in achieving optimal segmentation performance in these dimensions. To ameliorate this concern, researchers such as Schlemper et al. [26] have seamlessly integrated self-attention mechanisms into the framework of CNNs. In the pivotal year of 2017, Vaswani et al. [27] introduced the Transformer model, along with its inherent self-attention mechanisms, in the context of sequence-to-sequence tasks. This epochal development reverberated not only across the realm of natural language processing (NLP) but also found pertinence in the landscape of computer vision.

Buoyed by the transformative accomplishments of Transformers within NLP, a cohort of scholars has transposed this paradigm into the precincts of computer vision in recent years. Illustrative instances encompass the paradigm-shifting DETR [28] and UP-DETR [29] in the domain of object detection, the groundbreaking DeiT [30] in the realm of image classification, the pioneering CVT [31] for advancing face recognition, and the innovative PoseFormer [32] that has propelled human pose estimation to new frontiers. The watershed year 2020 witnessed Dosovitskiy et al. [33] ingeniously interweaving the prowess of Transformers into the tapestry of image classification, thereby engendering the seminal Vision Transformer (ViT) architecture. As the structural design of Transformers is inherently tailored for sequence data, whereas image segmentation pivots on two-dimensional canvases, ViT tactically partitions the image into distinct blocks. Subsequently, each block undergoes a transformation into a one-dimensional vector. To infuse the sequence with positional context, a technique hailing from NLP, known as positional encoding, is adroitly assimilated. The sequenced data are then channeled through an array of Transformer blocks, thereby effectuating feature extraction. Notably, unlike domains like machine translation, ViT dispenses with a dedicated decoder Transformer. Instead, the derived feature vectors are channeled directly into a multi-layer perceptron (MLP), culminating in the derivation of the image’s classification outcomes. Nurtured by this foundational innovation, a burgeoning spectrum of researchers have embarked upon the application of Transformers across diverse facets of image-related tasks.

For instance, Zhang et al. [34] adroitly amalgamate the conceptual ethos of the FPN with the transformative might of Transformers, birthing the ingenious FPF network architecture. This symbiotic alliance harnesses Transformers to orchestrate spatial feature interactions, while concurrently invoking FPN for orchestrating cross-scale feature interplay. In a parallel vein, Wang et al. [35] undertake a stratagem of hierarchical feature extraction, infusing Transformers endowed with spatially constrained attention at every stage of feature extraction. This dual-pronged maneuver adroitly encapsulates global contextual insights while concurrently tempering computational complexity. In the annals of 2022, the transformative TransUNet [36] emerges as a pioneering amalgamation of the canonical UNet architecture with the potency of Transformers, primed to surmount the nuanced challenges of medical image segmentation. Pioneering this trajectory, Hatamizadeh et al. [37] articulate Unetr, a visionary hybrid that marries the resounding efficacy of Transformers with the UNet framework, thus forging new frontiers in the domain of 3D medical segmentation.

This paper presents a novel approach for heart image segmentation using a pyramid-based architecture and a position-aware attention mechanism. The primary contributions and innovations of this work can be summarized as follows:

(1) Pyramid-based structure: This paper introduces a pyramid structure that draws inspiration from super-resolution reconstruction. By employing bicubic interpolation, the method generates multi-channel, multi-resolution medical images, which enhances the contextual information available for segmentation tasks. This approach effectively leverages the concept of image resolution enhancement to improve segmentation accuracy.
(2) Position-aware attention module: A Position Attention Block (PABlock) is proposed, integrating a position-aware attention mechanism. This module learns positional information in both horizontal and vertical directions and introduces position encoding. This attention mechanism enhances the model’s sensitivity to positional information during segmentation, leading to more accurate segmentation results.
(3) Experimental validation: The proposed method is rigorously validated through experiments on two distinct datasets: the EchoNet-Dynamic dataset and the ACDC dataset. The results demonstrate that the method achieves superior segmentation accuracy and competitiveness compared to other advanced methods.

## 2. Problem Description

In the progressive landscape of the contemporary medical practice, the relentless evolution of medical care has endowed medical imaging with an irreplaceable role as an essential adjunctive instrument in modern healthcare. Within this paradigm, the realm of medical image segmentation stands as a vanguard of profound research significance within the precincts of clinical medicine. The art and science of image segmentation, characterized by the partitioning of images into discrete and meaningful regions, augments the medical fraternity in multifarious dimensions. It empowers clinicians with the ability to engage in qualitative assessments of cellular tissues, pinpoints the precise localization of organ tissues, and facilitates quantitative evaluations of the intricate tapestry of human anatomical structures. However, the prevailing landscape reveals a noteworthy facet: the manual delineation and annotation of most medical images within healthcare institutions. This conventional approach hinges upon the specialized expertise of clinical professionals and demands an appreciable investment of temporal resources. Conspicuously, the exigencies of this manual methodology manifest in its labor-intensive nature, necessitating several laborious hours for the meticulous analysis of a solitary patient’s medical image. This temporal outlay, albeit meticulously employed, bespeaks a compelling imperative for transformative interventions in the realms of efficiency and expediency.

In the process of organ segmentation, an intriguing phenomenon emerges where clinicians instinctively direct their focus towards regions proximal to previously segmented areas. Analogously, this observation aligns with the conceptual underpinning of the attention mechanism within the realm of deep learning. Herein, the focal regions of significance within an image are endowed with augmented weight, thereby underscoring their importance, while regions bearing diminished information content are relegated to the background. In the broader context of organ segmentation, a prevailing consistency in the relative spatial disposition of the organs to be segmented prevails across a spectrum of medical images. Drawing from this inherent characteristic, it is plausible to surmise that the strategic integration of position-related information could impart substantive advantages to the enterprise of medical organ segmentation. Despite the palpable potential of this notion, it is noteworthy that contemporary methodologies for medical image segmentation have, for the most part, not embarked upon a trajectory that embraces this structural underpinning as a guiding principle.

Moreover, it is important to recognize that diverse medical images present a spectrum of resolutions. While the realm of segmentation predominantly encounters high-resolution medical images of pathological tissues, the scope of medical image segmentation extends beyond pathological tissue analysis to encompass routine clinical assessments. Consider, for instance, the realm of cardiovascular disease diagnosis and screening, wherein the precise determination of ejection fractions assumes paramount significance. The ejection fraction, a pivotal metric, hinges on the calculation of ventricular chamber volumes during diastole and systole phases of the cardiac cycle. Nonetheless, the ejection fraction calculation, typically confined to a finite cardiac cycle interval, often exhibits fluctuations. Consequently, the segmentation of the left ventricle from echocardiograms and the subsequent derivation of an average ejection fraction carry a substantive import. Nevertheless, it is imperative to recognize that echocardiogram resolutions pale in comparison to those of other medical images employed for anatomical localization and qualitative pathological tissue assessment. As a result, the resolution of medical images destined for quantitative analysis is markedly diminished, engendering a challenge in the extraction of comprehensive contextual information during the process of medical image segmentation. In light of prevailing circumstances, extant methodologies often endeavor to incorporate supplementary contextual insights by leveraging convolutional kernels of diverse scales during the phase of feature extraction. This strategy seeks to glean features spanning a gamut of scales and subsequently synthesize them. Nonetheless, in the context of medical images characterized by inherent low resolutions, the resultant features assume diminutive proportions, thereby engendering a dearth of extracted contextual nuances.

This paper presents a novel algorithm for segmenting heart images by leveraging the positional attention mechanism within an inverted pyramid framework. The methodology enriches the baseline model by incorporating a multi-scale fused inverted pyramid structure and a Position Attention Block rooted in the principles of positional attention. Drawing inspiration from the realm of image super-resolution reconstruction, the inverted pyramid mechanism employs multi-channel, multi-resolution medical images as inputs, thereby harnessing a wealth of contextual information. The Position Attention Block, through the introduction of positional encoding (Position Embedding), imparts an intensified focus on positional information during the segmentation process, resulting in an elevated accuracy of network-based segmentation. Experimental findings unequivocally demonstrate the superiority of the proposed technique, surpassing alternative advanced methodologies in terms of segmentation accuracy across two distinct datasets dedicated to the segmentation of cardiac ventricles.

## 3. Method

In light of the prevalence of low-resolution medical images and the consistent spatial positioning of medical organs within the overall image, it becomes imperative to empower neural networks to glean enhanced contextual insights from these lower-resolution medical images during the process of medical segmentation. Moreover, a concerted focus on the positional attributes of medical organs assumes paramount importance. In this paper, we put forth a medical image segmentation algorithm that rests upon the foundation of a positional attention mechanism synergistically integrated with an inverted pyramid structure. The introduced inverted pyramid structure effectuates a transformation of the initial network architecture into a multi-scale configuration, adeptly catering to the necessity of encapsulating contextual nuances spanning various scales. Furthermore, we introduce a novel PABlock meticulously designed to capture and harness positional information. Within the network’s operational workflow at each scale, the employment of three successive PABlocks serves to amplify the infusion of positional information, thereby conferring a tangible augmentation upon the performance of the segmentation task.

### 3.1. The Overall Network Framework

**Figure 1:**
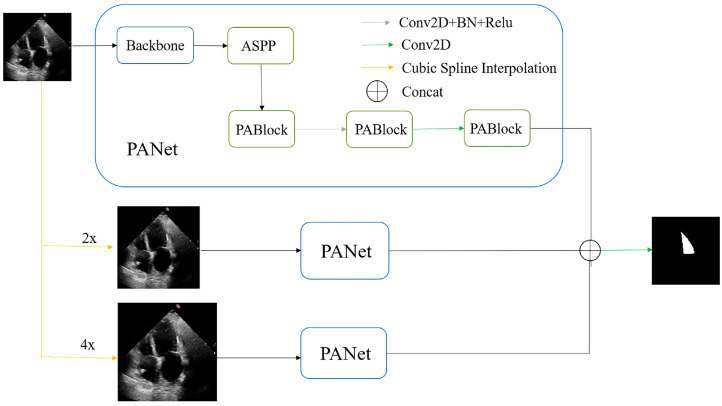
Overall framework of medical image segmentation using position attention-based inverted pyramid structure.

The schematic depiction of the proposed method’s comprehensive framework is illustrated in Figure 1. The procedural initiation commences with input images, which subsequently undergo a transformation to generate three distinct images characterized by varying scales. These images are then channeled into the training network through three distinct channels. The pivotal orchestrator in this framework is the Position Attention Network (PANet), which is meticulously designed to incorporate position-oriented attention mechanisms.

Each individual PANet undertakes the processing of its respective input image, adhering to a specific resolution scale. The PANet’s output materializes as the anticipated segmentation outcome at that specific scale. The PANet’s deployment serves as a conduit for the seamless amalgamation of positional intelligence and contextual cognizance into the segmentation endeavor. This amalgamation empowers the network to engender segmentation outputs of utmost precision by leveraging the distinctive attributes unique to each scale.

Each PANet within the training network, across diverse scales, is meticulously constructed upon the foundational architecture of ResNet101’s Deeplab v3 network. Within this network framework, ResNet101 serves as the bedrock, extracting features spanning a gamut of scales—from rudimentary to sophisticated, from concrete to abstract—through an assemblage of five successive convolutional strata. Following this feature extraction, the Atrous Spatial Pyramid Pooling (ASPP) module takes the reins. The ASPP harnesses atrous convolutions characterized by distinct rates, fostering an expansion of the receptive field across multifarious scales, thereby encapsulating contextual information that is multifaceted in scale. Eventually, the feature maps navigate through a trinity of PANet modules, wherein the infusion of positional information into the feature maps is executed. The predictions wrought at the three discrete scales culminate in concatenation, and the final outcome is computed in accordance with Equation (1).

(1) $$y=\mathrm{conv}(\mathrm{concat}\left(y_{1},y_{2},y_{3}\right))$$

In this equation, *y* signifies the ultimate predicted image, while $y_{1}$, $y_{2}$, and $y_{3}$ correspond to the resultant prediction images at the three distinct scales.

### 3.2. Inverted Pyramid Structure

Owing to the inherent divergence in data acquisition apparatuses and settings, coupled with the subjective variations in the aims underlying image procurement, the resultant medical images invariably exhibit a spectrum of resolutions. Notably, image resolution plays an instrumental role in dictating the capability of images to faithfully convey the nuances of objects. In the realm of two-dimensional medical images, the dimensions of spatial resolution and color fidelity assume paramount importance. Images endowed with elevated resolution typically encapsulate a profusion of intricate details and textural intricacies, alongside a bounty of contextual insights, which invariably translates into an ameliorated state of segmentation outcomes. Nonetheless, procuring high-resolution images directly might entail escalated costs, thus prompting an exploration of algorithmic methodologies from the vantage point of software, which stand to be inherently more cost-effective. Against this backdrop, this paper unfolds the introduction of an inverted pyramid structure, which is a strategic apparatus that orchestrates the elevation of lower spatial resolution images to the echelons of higher resolution, all in the service of segmentation. This resolute elevation engenders the infusion of augmented contextual acumen, thereby heralding an augmentation in the performance of segmentation endeavors.

The inverted pyramid structure introduced within this paper aligns itself with the realm of multi-scale fusion strategies, a domain characterized by its adeptness in seamlessly amalgamating low-level attributes with intricate particulars, alongside high-level characteristics intertwined with all-encompassing insights. Within the precincts of image segmentation, the panorama of multi-scale fusion strategies typically assumes the form of early and late fusion paradigms contingent upon the sequence governing the fusion and prediction stages. The echelons of early fusion are characterized by the extraction of features across disparate scales, which is a task adroitly executed by distinct module architectures during the network’s tutelage. Subsequently, these manifold features are harmoniously fused, oftentimes through techniques like residual structures or skip connections. In contradistinction, the corridors of late fusion entail the rescaling of images to divergent dimensions prior to the onset of training. This process begets a manifold of image channels, each delineating a distinct scale, thereby enabling the network to train and prognosticate within the confines of these distinctive channels. The culminating stage witnesses the amalgamation of prognostic outcomes hailing from this panoply of channels. Nonetheless, the existing tapestry of late fusion strategies within the image segmentation stratum predominantly gravitates towards assimilating all-encompassing insights from high-level attributes; this is a course of action that invariably triggers the downsizing of the native images. However, when it comes to the realm of low-resolution images, the quest for assimilating augmented contextual perspicacity mandates the accession of a greater repertoire of low-level attributes. This juxtaposition imparts an inherent inadequacy to the time-honored late fusion strategies in effectively navigating such scenarios.

The conceptual framework of the inverted pyramid structure, introduced within this paper, derives its impetus from the realm of image super-resolution reconstruction. It orchestrates an elevation in the resolution of the original input image, subsequently embarking on parallel training across diverse scales of image channels. This intricate orchestration begets separate prediction outcomes, which convene in a subsequent synthesis to amplify the reservoir of local information. Remarkably, the inverted pyramid structure capitalizes on the venerable paradigm of traditional bicubic interpolation-based super-resolution reconstruction. This strategic selection arises due to the exigencies inherent in medical image analysis. The conventional deep learning-based super-resolution methods typically necessitate high-resolution ground truth data for effective training, a requirement often infeasible in the domain of medical images characterized by their low-resolution counterparts. Within this context, the traditional bicubic interpolation method is harnessed to extend its prowess, augmenting image dimensions and harnessing known pixel information to infer hitherto unknown pixel values.

To delve into the intricacies of the process, consider an input image denoted as $X\in R^{c\times h\times w}$, where h×w signifies the spatial resolution of the image and *c* corresponds to the image’s channel dimension. The latter typically takes on the value of 3 or 1, corresponding to color and grayscale images, respectively. Now, imagine the scenario where image *X* requires an enlargement to a distinct size; let us say $F\in R^{c\times 2h\times 2w}$. In this endeavor, it becomes imperative to conjure up the pixel values for the expanded image *F* based on the existing pixel data. To fulfill this task, the method of choice is the bicubic interpolation technique.

**Figure 2:**
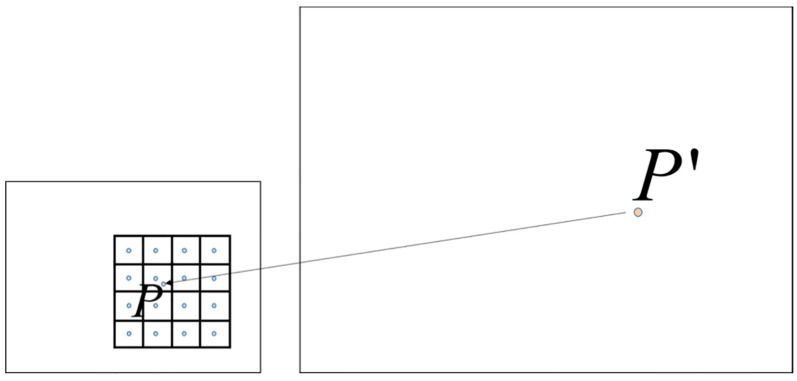
Example illustration of mapping unknown pixel *P* in bicubic interpolation of the original image.

Consider, for example, the task of determining the value of an uncharted pixel $P^{{}^{\prime }}$ following an image’s magnification. In this context, the bicubic interpolation technique comes into play. This technique commences by first aligning the unknown pixel with its counterpart in the original-sized image, leveraging the scaling ratio for guidance. This alignment yields the exact coordinates P(x,y) in the initial image. With this location established, the value of the enigmatic pixel $P^{{}^{\prime }}$ is meticulously calculated. This computation draws upon the values of the nearest 16 neighboring pixels $Q_{i,j}\left(x_{i,j},y_{i,j}\right)$ encircling the pivotal pixel P(x,y), as visually depicted in Figure 2. In this visual representation, the subscripts *i* and *j* span from 1 to 4, encompassing distinct positions around the central pixel P(x,y).

The utilization of bicubic interpolation to approximate unknown pixel values constitutes a pivotal process in image super-resolution reconstruction. This process empowers the expansion of images by drawing on the insights from surrounding pixel data, culminating in the creation of higher-resolution renditions of the images. The methodology encompasses a judicious amalgamation of neighboring pixel values, orchestrating the derivation of the enigmatic pixel values. The outcome is a heightened degree of refinement and visual allure in the image enlargement endeavor, in contrast to more rudimentary techniques such as nearest-neighbor interpolation.

Bicubic interpolation computes the pixel value at point *P* within the target image based on the image scaling factor, as demonstrated in Equation (2):

(2) $$P\left(x,y\right)=\sum_{i=1}^{4}\sum_{j=1}^{4}Q_{ij}\times W\left(i\right)\times W\left(j\right)$$

Here, P(x,y) signifies the pixel value under determination, $Q_{i,j}$ denotes the values attributed to the 16 neighboring pixels, and W(i) and W(j) symbolize the corresponding weight coefficients. These weight coefficients encapsulate the relational coefficients with regards to the spatial distance between the known pixel $Q_{i,j}$ and P across two dimensions. The derivation of W() is derived from the bicubic function, explicitly formulated in Equation (3):

(3) $$W\left(x\right)=\left\{\begin{array}{ccc} {\left(a+2\right)|x|}^{3}-\left(a+3\right){\left|x\right|}^{2}+1, & \; & |x|\leq 1\\ {a|x|}^{3}-{5a|x|}^{2}+8a\left|x\right|-4a, & \; & 1<|x|<2\\ 0, & \; & others \end{array}\right.$$

In this context, W(x) stands as the weight coefficient corresponding to the computation of P(x,y) concerning the alternative pixel $Q_{i,j}$, whereby *x* represents the vertical or horizontal separation between $Q_{i,j}$ and P, and *a* embodies a hyperparameter commonly assigned a value of −0.5.

### 3.3. Position Attention Block (PABlock)

During the segmentation process at each scale, the segmentation is carried out by the PANet, an attention-based network. Within the PANet, the Position Attention Block (PABlock) is introduced to bolster segmentation accuracy through an increased emphasis on position information. Position Embedding is integrated into the PABlock to steer the network’s attention towards position-related particulars during the segmentation process.

**Figure 3:**
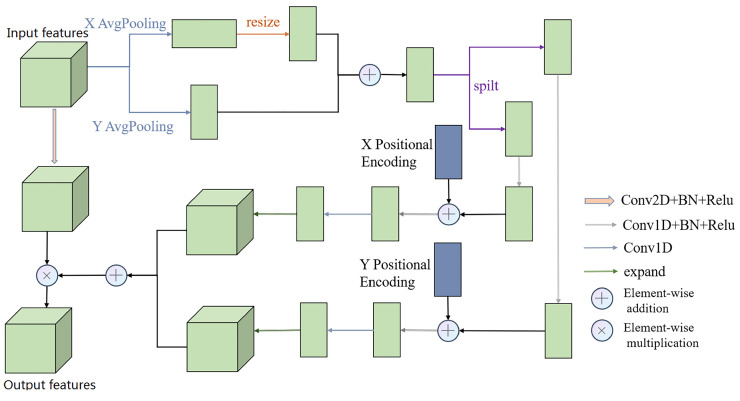
The structure of the Position Attention Block (PABlock).

The specific structure of the PABlock is depicted in Figure 3. This architecture empowers the network to adeptly capture and harness position-specific cues within medical images, consequently enhancing the precision of the segmentation outcomes.

(1) Spatial Dimension Pooling

To derive an attention weight matrix that accounts for position, it is imperative to accumulate contextual insights concerning height for each row element within the input features and analogous insights regarding width for each column element. In a more detailed manner, given an input matrix $X\in R^{c\times h\times w}$, distinct pooling operations are conducted based on width and height, culminating in matrices that encapsulate height-related and width-related insights. Among the various pooling operations such as max-pooling and average pooling, the latter is selected based on empirical knowledge. Subsequently, the height-related matrix captures extensive dependencies across the horizontal dimension of the input matrix, concurrently conserving accurate positional particulars along the vertical axis. The resultant vector within the *h*-th row of the height-related matrix can be represented as exemplified in Equation (4):

(4) $$Z_{width-pooling}{\left(X\right)}_{:,h}=\left[\frac{1}{W}\sum_{i=1}^{W}X_{1,h,i};\ldots ;\frac{1}{W}\sum_{i=1}^{W}X_{C,h,i}\right]$$

Here, $Z_{width-pooling}$ signifies width-oriented average pooling, $X\in R^{C\times h\times w}$ denotes the input feature matrix, *h* signifies the index of the acquired height-based matrix row, and *C*, *H*, and *W* symbolize the dimensions of the input matrix *X*.

Analogously, the operation of pooling width-wise generates a matrix enriched with width-related insights, achieved by aggregating each column element within the input features. Through this process, extensive dependencies across the vertical axis of the input matrix are captured, maintaining accurate positional particulars along the horizontal axis. The column vector corresponding to the w-th column of the resulting width information matrix can be mathematically expressed as depicted in Equation (5):

(5) $$Z_{height-pooling}\left(X\right)w;:=\left[\frac{1}{H}\sum_{i=1}^{H}X_{1,i,w};\ldots ;\frac{1}{H}\sum_{i=1}^{H}X_{C,i,w}\right]$$

Here, $Z_{height-pooling}$ denotes height-based average pooling, $X\in R^{C\times h\times w}$ signifies the input feature matrix, *w* signifies the index of the obtained width-related matrix row, and *C*, *H*, and *W* symbolize the dimensions of the input matrix *X*.

For the purpose of merging these two matrices, a transformation is applied to the height information matrix to align it with the dimensions of the width information matrix. Consecutively, the amalgamation of the two matrices yields a feature map infused with meticulous positional insights. This fusion procedure is graphically exemplified in Equation (6):

(6) $$Z=conv(concat\left(Z_{height-pooling}\left(X\right),Z_{width-pooling}\left(X\right)\right))$$

Here, *Z* signifies the resulting feature map enriched with seamlessly fused, precise positional insights. $Z_{height-pooling}\left(X\right)$ and $Z_{width-pooling}\left(X\right)$ pertain to the previously derived width information matrix and height information matrix, respectively. The notation “conv” embodies a sequence of one-dimensional convolution operations followed by normalization and ReLU activation.

Subsequent to the pooling of spatial dimensions, the resultant feature map matrix $Z\in R^{C\times 1\times (H+W)}$, encompassing amalgamated meticulous positional information, undergoes partitioning into two distinct matrices: $Z_{height}\in R^{C\times 1\times H}$ and $Z_{width}\in R^{C\times 1\times W}$. This division is conducted to enable the integration of supplementary positional encoding information into the feature matrix across both spatial orientations.

(2) Spatial Compression

Before incorporating the positional encoding information, not all rows or columns of the features bear equal significance when computing positional weights. Drawing inspiration from the concept of SENet [20], a downsampling operation with a ratio of r=64 is applied to the previously derived $Z_{height}\in R^{C\times 1\times H}$ and $Z_{width}\in R^{C\times 1\times W}$ matrices for spatial compression. Following the integration of positional encoding information, the acquisition of a positional weight matrix with the same dimensions as the input matrix necessitates a resizing process and a dimensional transformation to align with the dimensions of the attention matrix.

(3) Positional Encoding Embedding

After obtaining the downsampled features $Z_{height}\in R^{C\times 1\times H/r}$ and $Z_{width}\in R^{C\times 1\times W/r}$, the PABlock incorporates prior knowledge about foreground information in both horizontal and vertical directions by introducing positional encoding information from the field of NLP. In NLP, positional encoding information comes in two forms: absolute encoding and relative encoding. For the context of image segmentation, the network must learn the relative relationships between pixels in different rows or columns along both the horizontal and vertical directions. Thus, relative positional encoding information is employed.

In particular, the sine positional encoding, widely used in NLP, is adopted. This involves applying trigonometric sine and cosine functions to transform positional encoding information between different pixels, thereby capturing their relative relationships. The computation of positional encoding information associated with height (Height Positional Encoding, HPE) in this module is represented by Equation (7):

(7) $$HPE\left(h,i\right)=\left\{\begin{array}{ccc} sin(\frac{h}{1000^{\frac{i}{d}}}), & \; & i=2k\;(k=1,2,3,\ldots )\\ cos(\frac{h}{1000^{\frac{i-1}{d}}}), & \; & i=2k+1\;(k=1,2,3,\ldots ) \end{array}\right.$$

Here, *h* denotes the height position within the HPE matrix, and *i* represents the dimension, where i∈1,…,d and d=512. Similarly, the computation of positional encoding information related to width (Width Positional Encoding, WPE) is outlined in Equation (8):

(8) $$WPE\left(h,i\right)=\left\{\begin{array}{ccc} sin(\frac{w}{1000^{\frac{i}{d}}}), & \; & i=2k\;(k=1,2,3,\ldots )\\ cos(\frac{w}{1000^{\frac{i-1}{d}}}), & \; & i=2k+1\;(k=1,2,3,\ldots ) \end{array}\right.$$

Here, *w* represents the width position within the WPE matrix, and *i* denotes the dimension, with i∈1,…,d and d=512.

(4) Feature Fusion

Upon obtaining the matrix enriched with embedded positional encoding information, the restoration process to match the original dimension size is imperative for the subsequent fusion of the weight matrix with the input features. The process of reinstating the feature matrix along any direction is expressed through Equation (9):

(9) $$O=Expand(conv\left(G_{UP}\left(concat\left(PE,Z_{down}\right)\right)\right))$$

Here, *O* denotes the feature acquired after restoring the corresponding dimension size along any given direction. The operation Expand involves replicating the vector in the respective direction, either for the original height information matrix with dimensions C×H×1 or the width information matrix with dimensions C×1×W, thus expanding it to C×H×W. The term conv signifies an 11 one-dimensional convolution, while $G_{UP}$ encompasses a series of operations: upscaling with a factor of r=64, followed by an 11 one-dimensional convolution, normalization, and ReLU activation.

Subsequently, the positional information matrices obtained from both directions are merged through element-wise addition and convolution operations. This amalgamated information is then employed within a residual module to conduct a weighted operation on the input matrix of the PABlock, yielding the final PABlock output.

Empirical insights advocate for the arrangement of three consecutive PABlocks within the PANet architecture. The initial PABlock takes the feature extracted by the ASPP module as input, while the subsequent two PABlocks receive outputs from their preceding counterparts. Furthermore, the output of the ultimate PABlock necessitates passage through a segmentation head to generate the network’s segmentation predictions. The computation process of the segmentation head is outlined in Equation (10):

(10) $$Output=G_{UP}\left(conv\left(F\right)\right)$$

Here, Output signifies the ultimate predicted segmentation outcome, $G_{UP}$ represents the operation for restoring resolution through upscaling, conv denotes a two-dimensional convolution operation, and *F* corresponds to the feature map output from the final PABlock.

## 4. Experiments and Analysis

### 4.1. Datasets and Evaluation Metrics

The proposed approach detailed in this paper underwent comprehensive evaluation using two publicly available datasets specifically designed for cardiac ventricle segmentation. The first dataset employed is the EchoNet-Dynamic dataset [38]. This dataset encompasses a vast collection of 10,030 echocardiographic videos of the cardiac apex, obtained through routine clinical procedures at Stanford Medical School. Originally curated for the computation of cardiac ejection fraction, each video within the dataset corresponds to a patient’s clinical visit record. In the context of this study, end-diastolic and end-systolic frames were meticulously extracted from each video, effectively creating a dataset of 20,060 left ventricular echocardiographic frames. These frames are characterized by a resolution of 112 × 112 pixels and have been manually annotated by expert sonographers and cardiologists. In further detail, the extracted images were methodically divided into distinct subsets for training, validation, and testing. These subsets contain 14,930, 2554, and 2576 images, respectively, facilitating a robust and comprehensive evaluation of the proposed methodology.

The supplementary dataset utilized in this study is the Automatic Cardiac Diagnosis Challenge (ACDC) dataset [39]. This dataset emanates from the 2017 MICCAI-sponsored automatic cardiac diagnosis challenge. It encompasses a diverse range of segmentation targets, including the right ventricle, left myocardium, and left ventricular cavity. Derived from a cohort of 100 patients, each providing two distinct time frames of cardiac images within a cardiac cycle, the ACDC dataset predominantly comprises MRI data, with each MRI scan corresponding to a unique patient. To facilitate the research objectives of this study, a careful selection process was employed, extracting images from 70 MRI cases for the training set, 20 for the validation set, and 10 for the testing set. The specific task associated with this dataset involves predicting the segmentation of the left ventricular cavity. To ensure consistent conditions, all MRI images were initially preprocessed into a standardized resolution of 112×112 pixels in the form of 2D images.

The assessment of agreement between the predicted segmentation outcomes and the ground truth in this medical image segmentation study is carried out using the Dice similarity coefficient and the Jaccard coefficient (Jc). These evaluation metrics yield values within the 0 to 1 range, with higher scores denoting increased congruence between the predicted and segmented regions, thereby indicating enhanced prediction accuracy. The formulas for calculating the Dice similarity coefficient and Jaccard coefficient are presented in Equations (11) and (12) as follows:

(11) $$Dice=\frac{2|R_{pred}\cap R_{gt}|}{|R_{pred}\left|+\right|R_{gt}|}$$

(12) $$Jc=\frac{|R_{pred}\cap R_{gt}|}{|R_{pred}\left|\cup \right|R_{gt}|}$$

where $R_{pred}$ signifies the predicted segmentation outcome, and $R_{gt}$ denotes the ground truth segmentation outcome.

Moreover, segmentation methods often necessitate the evaluation of results for both true and false samples. This paper’s evaluation criteria encompass Precision, Recall, and F-Measure. Precision quantifies the proportion of accurately predicted pixels in the segmentation result to the total predicted pixels. Recall gauges the ratio of correctly predicted pixels to the total ground truth pixels, offering insight into the algorithm’s capability to correctly identify regions of interest. These evaluation metrics, like the previously mentioned ones, adopt values from 0 to 1, where elevated values signify superior performance of the segmentation algorithm. Their calculation formulations are detailed in Equations (13) and (14):

(13) $$Precision=\frac{TP}{TP+FP}=\frac{|R_{gt}\cap R_{pred}|}{R_{pred}}$$

(14) $$Recall=\frac{TP}{TP+FN}=\frac{|R_{pred}\cap R_{gt}|}{R_{gt}}$$

Here, TP signifies the count of pixels accurately predicted as part of the segmentation target; FP indicates the number of pixels falsely predicted as background; FN represents the number of pixels falsely predicted as the non-segmentation area; $R_{pred}$ and $R_{gt}$ possess the same meanings as defined in Equation (12).

Nevertheless, Precision and Recall frequently interact, often making it challenging to maximize them simultaneously. Consequently, during the assessment of network performance, a balance between these two metrics is imperative. Thus, the F-Measure metric is commonly adopted to provide a comprehensive evaluation, accounting for both Precision and Recall. A higher F-Measure score denotes superior method performance. The F-Measure calculation is expressed in Equation (15):

(15) $$F-\mathrm{Measure}=\frac{(a^{2}+1)\times \mathrm{Precision}\times \mathrm{Recall}}{a^{2}\times \left(\mathrm{Precision}+\mathrm{Recall}\right)}$$

In this equation, Precision and Recall, respectively, pertain to accuracy and recall metrics; a represents a parameter harmonizing Precision and Recall, which is conventionally set to 1.

### 4.2. Experimental Environment

The method presented in this paper is implemented using the PyTorch deep learning framework, and all experiments are carried out on an NVIDIA RTX 2080Ti GPU with 11 GB of memory. The initial learning rate for training is configured at 0.01, and the training process employs the SGD optimizer with a momentum value of 0.9. The learning rate is subjected to an equal interval reduction strategy. The model undergoes training for a cumulative 150 epochs across the complete dataset, employing a batch size of 8 for training images.

### 4.3. Experiments Related to the Inverted Pyramid (IP) Structure

In conventional multi-scale training approaches for image segmentation, the typical strategy to extract more contextual information involves conducting feature extraction followed by downsampling. This yields images or features with reduced resolutions, which are then input into multi-channel networks for training. The primary aim of this method is to acquire high-level abstract features at lower resolutions during training and fuse them with low-level semantic features at the original resolution. The intention is to gather more comprehensive contextual information. In this section, a set of experiments was conducted to assess the impact of scale choices in multi-scale training, comparing both the conventional approach and the concept of image super-resolution reconstruction. The baseline model used for comparison is Deeplab v3.

Moreover, the Inverted Pyramid (IP) structure, drawing inspiration from image super-resolution reconstruction, encompasses various techniques including traditional methods such as bilinear and bicubic interpolation, as well as more advanced deep learning-based reconstruction methods like LapSRN [40]. To evaluate the performance of these methods, this section compares traditional image methods with deep learning-based techniques that employ LapSRN for image reconstruction to attain higher resolutions. Given the inherent scarcity of higher-resolution images in low-resolution medical images, the LapSRN reconstruction network utilizes upsampled versions of the input images as pseudo high-resolution images for supervision.

The specific outcomes of the experiments are detailed in Table 1. The data in the table reveal that multi-scale networks with ratios of [1x,0.5x,0.25x] using bicubic interpolation outperform the baseline across all five evaluation metrics. However, the performance of multi-scale networks with ratios of [1x,2x,4x] is even more superior. In particular, the [1x,2x,4x] results exhibit an improvement of 3.43% in Dice, 8.34% in Jc, 2.05% in Precision, 3.41% in Recall, and 2.72% in F-Measure compared to [1x,0.5x,0.25x]. Consequently, it can be deduced that for low-resolution medical images, the approach of image super-resolution reconstruction, which allows for capturing more contextual information during network training, outperforms the conventional approach of fusing low-level semantic features from high resolution with high-level abstract features from low resolution, leading to improved performance.

Furthermore, a thorough analysis of the data in the table reveals an interesting pattern: despite both methods aiming to reconstruct the original images to the same [1x,2x,4x] scale, the segmentation outcomes obtained through bicubic interpolation consistently outperform the results achieved through LapSRN image reconstruction across all five evaluation metrics: Dice, Jaccard coefficient (Jc), Precision, Recall, and F-Measure. To elaborate, the segmentation experiment outcomes using bicubic interpolation demonstrate improvements over the LapSRN results in the following aspects: a 0.85% enhancement in Dice, a 2.23% improvement in Jc, a 0.16% enhancement in Precision, a 3.29% improvement in Recall, and a 1.71% enhancement in F-Measure.

These findings lead to the conclusion that in the context of applying the concept of image super-resolution reconstruction to segment low-resolution images, especially in scenarios where high-resolution image supervision is not available, the segmentation performance achieved through bicubic interpolation of the reconstructed images surpasses the performance achieved through LapSRN image reconstruction. The selection of bicubic interpolation for image reconstruction in our study was driven by its simplicity, efficiency, and common usage as a baseline method in image super-resolution tasks. Bicubic interpolation serves as a straightforward and widely employed method for upscaling images, providing a basis for comparison with more sophisticated techniques. While it may lack the complexity of deep learning-based methods, its inclusion allows us to establish a performance baseline and evaluate the efficacy of our proposed method against a simpler yet prevalent approach.

It is important to note that the LapSRN approach for image reconstruction demands additional training time to generate higher-resolution images and also requires additional storage space to accommodate these images. Furthermore, the reconstructed high-resolution images need to be integrated into the segmentation network for further processing. In contrast, the application of bicubic interpolation can be directly incorporated into the image preprocessing phase of the segmentation network. Consequently, when contemplating the utilization of image super-resolution reconstruction for segmenting low-resolution images, opting for bicubic interpolation not only yields superior segmentation performance and adopts a simpler approach, but it also necessitates less time and storage space compared to the LapSRN method.

### 4.4. Ablation Experiments

In contrast to existing methodologies, the approach introduced in this paper is inspired by the concept of image super-resolution reconstruction. It employs bilinear upsampling to magnify low-resolution medical images, forming a reversed pyramid structure. This innovation allows for the utilization of multi-scale images as inputs during network training, consequently enabling the capture of a richer contextual understanding. Moreover, recognizing the significance of prior location information in medical organ images, this paper introduces the Position Attention Block (PABlock) coupled with embedded positional encoding. This addition enhances the network’s focus on acquiring spatial information within images. In order to evaluate the collective impact of these two advancements on the overall model, this section conducts ablation experiments on the EchoNet-Dynamic dataset.

In the ablation experiment involving the pyramid structure, the input images undergo an enlargement process using bicubic interpolation, resulting in images with 1*x*, 2*x*, and 4*x* dimensions of the original input image. Subsequently, these resized images are introduced into the multi-scale network. Regarding the ablation experiment related to the Position Attention Block (PABlock), the PABlock is employed in succession three times following the Atrous Spatial Pyramid Pooling (ASPP) module, a configuration determined through empirical observations. Both ablation experiments utilize the Deeplab v3 architecture with a ResNet101 backbone as the baseline. The outcomes of these two enhancement ablation experiments are summarized in Table 2.

Based on the information presented in Table 2, both the proposed reverse pyramid structure and the inclusion of the Position Attention Block (PABlock) exhibit enhancements across all five evaluation metrics. Notably, the reverse pyramid structure leads to a notable increase of 2.75% in Dice, 4.3% in Jc, 7.68% in Precision, 4.61% in Recall, and a substantial 6.25% improvement in F-Measure. Similarly, the application of the PABlock contributes to improvements of 1.87% in Dice, 2.81% in Jc, 9.57% in Precision, 0.88% in Recall, and a significant 5.39% enhancement in F-Measure. These outcomes effectively underscore that each of these enhancements independently plays a significant role in augmenting the performance of the segmentation algorithm.

Moreover, when both the reverse pyramid structure and PABlock are applied simultaneously in the ablation experiments, the results surpass the individual improvements achieved by each structure alone. Across the Dice, Jc, Precision, and F-Measure metrics, this combined approach yields superior results. While the Recall metric in this combined scenario experiences a marginal 0.44% decrease from its peak value, it is important to acknowledge that achieving the highest Recall and Precision values simultaneously is often challenging. The F-Measure, being a comprehensive metric that considers both Recall and Precision, is of utmost importance for evaluating overall performance. This outcome confirms the effectiveness of the proposed approach.

These findings suggest that the two structures, though enhancing the network’s performance in distinct ways compared to the baseline, also synergize effectively. Although the ablation experiments demonstrate significant advancements in segmentation performance, it’s crucial to note that these results do not inherently imply the superiority of the proposed method over other established techniques. Further comparative assessments are necessary to make such claims.

### 4.5. Comparative Experiments with Existing Methods

To provide a more comprehensive illustration of the enhanced segmentation performance of our proposed method relative to other approaches, we carried out comparative experiments on both the EchoNet-Dynamic dataset and the ACDC dataset. The detailed results of these comparative experiments on the EchoNet-Dynamic dataset and the ACDC dataset are presented in Table 3 and Table 4, respectively.

To ensure the fairness of the experiments, all methods were evaluated using identical data preprocessing, parameter configurations, and training environments. For the advanced methods HANet [44] and CABlock [22], which are effective attention modules, we ensured uniform conditions by integrating three consecutive attention modules after the ASPP module of Deeplab v3. Subsequently, segmentation heads were employed for the segmentation process. El Rai et al. [45] introduced a semi-supervised segmentation approach for echocardiography videos using graph signal processing. While it addresses certain challenges, our proposed method exhibits competitive performance, particularly in terms of segmentation accuracy and efficiency. Fan et al. [46] proposed ViT-FRD, a vision transformer model, focusing on cardiac MRI image segmentation based on feature recombination distillation. Our method demonstrates comparable or superior performance, highlighting its effectiveness across different imaging modalities. Farhad et al. [47] presented cardiac phase detection in echocardiography using convolutional neural networks. Our approach outperforms this method in terms of segmentation precision, showcasing its potential for more accurate delineation of cardiac structures.

As illustrated in Table 3, our proposed method surpasses other approaches across various evaluation metrics, including Dice, Jc, Recall, and F-Measure. Notably, our method exhibits a 0.78% higher Dice score compared to the second-best result (DUNet), a 4.95% higher Jc score, a 0.78% higher Recall score, and a 0.44% higher F-Measure score. While our method’s Precision score is 0.78% lower than the top-performing result (DANet), the balance between Recall and Precision becomes evident in the F-Measure, where our approach excels. In terms of the comprehensive evaluation metrics, our proposed method outperforms other advanced techniques.

**Figure 4:**
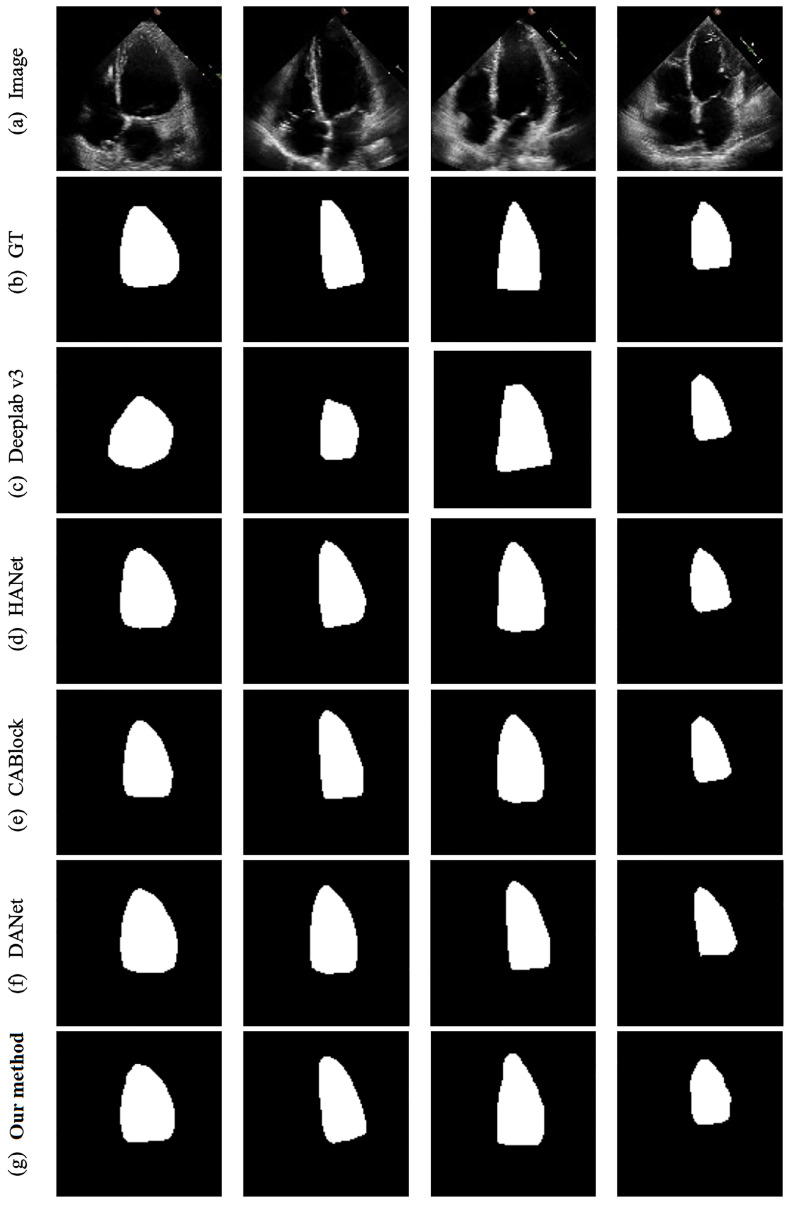
Visual results of comparative experiments with other advanced methods on the EchoNet-Dynamic dataset.

Based on the objective experimental findings using the EchoNet-Dynamic dataset, and further supported by the visualized segmentation outcomes displayed in Figure 4, it becomes evident that our proposed method excels. In this comparison, the baseline is established by using Deeplab v3. Upon examining the visualized results of Deeplab v3, a notable divergence between its predictions of the heart ventricle’s positional distribution and the ground truth is discernible. This observation implies that the baseline model does not sufficiently emphasize location information within the images. In contrast, our method’s predictions display superior performance in accurately capturing the positional distribution of the heart ventricle compared to Deeplab v3. This enhancement is attributed to the incorporation of position-based attention modules that amalgamate features from both horizontal and vertical orientations while integrating position matrices in each direction. Consequently, the network becomes adept at prioritizing spatial relationships between pixels, leading to the improved segmentation outcomes.

Upon contrasting our approach with advanced techniques like HANet, CABlock, and DANet, it becomes apparent that although these methods do take position-related information into account, they encounter difficulties when it comes to capturing intricate details within the confines of low-resolution images. Our method, in comparison, excels in providing more precise predictions in localized regions. This distinction underscores the efficacy of harnessing the inverted pyramid structure to amass a richer reservoir of contextual information.

In the comparison between PABlock, CABlock, and HANet, the experimental outcomes demonstrate that employing only PABlock yields the most favorable results across the Dice, Jc, and Recall metrics. Notably, in terms of the Dice metric, PABlock exhibits a superiority of 0.87% over CABlock and 0.77% over HANet. Similarly, regarding the Jc metric, PABlock outperforms CABlock and HANet by 1.34% and 0.63%, respectively. In the context of the Recall metric, PABlock showcases an advantage of 0.54% over CABlock and 1.39% over HANet. Despite PABlock having a Precision metric lower than that of HANet, considering the comprehensive F-measure, PABlock emerges as the most effective, surpassing CABlock and HANet by 0.77% and 0.37%, respectively.

Furthermore, our approach’s performance on the ACDC dataset, which revolves around left ventricle segmentation, is also subjected to evaluation. Luo et al. [48] proposed a semi-supervised heart image segmentation approach through dual-task consistency. While this method addresses certain challenges, our approach achieves superior results on the ACDC dataset, emphasizing its effectiveness in left ventricular cavity segmentation. Wu et al. [49] proposed a semi-supervised left atrium segmentation with mutual consistency training, focusing on specific cardiac structures. In our comparison, our method demonstrates competitive or superior performance, showcasing its versatility across different segmentation targets. Wu et al. [50] explored smoothness and class-separation for semi-supervised heart image segmentation. Our method excels in terms of segmentation accuracy, underscoring its effectiveness in handling diverse segmentation tasks. As indicated in Table 4, our method secures the highest rankings across the Dice, Jc, Recall, and F-Measure metrics. Specifically, our method outpaces the second-best outcome (Deeplab v3) by 4.04% in Dice, 2.18% in Jc, 4.02% in Recall, and 3.18% in F-Measure. Despite our Precision score lagging behind the best (DUNet) by 1.56%, the amalgamation of Recall and Precision in the F-Measure still highlights our method’s strong performance.

In the realm of modern medical imaging and data analysis, the pivotal role of sensors cannot be understated. Our research demonstrates the symbiotic relationship between sensor technology and advanced image segmentation methodologies. Sensors serve as the cornerstone for capturing essential data, including vital signs, anatomical features, and physiological parameters. In our study, the data obtained from sensors are integral in enhancing the precision and efficiency of cardiac image segmentation. These sensors provide critical inputs that enable our model to learn and adapt, resulting in superior segmentation accuracy. Furthermore, sensors facilitate the continuous monitoring of patients, enabling real-time adjustments and interventions based on segmented data. The integration of sensor-generated data with cutting-edge machine learning techniques represents a paradigm shift in medical imaging. It not only streamlines the diagnostic process but also opens new avenues for personalized and data-driven healthcare. As sensor technologies continue to advance, their synergy with medical imaging will lead to transformative innovations, ultimately benefiting both patients and healthcare providers.

Considering the comprehensive analysis of the objective experimental results on both the EchoNet-Dynamic and ACDC datasets, along with the visualized segmentation outputs, it becomes clear that our proposed method showcases advancements over existing mainstream techniques.

While this research contributes significantly to the domain of heart image segmentation, there are avenues for future exploration and enhancement. The noted resemblance to previous work underscores the importance of conducting a more extensive comparative analysis, elucidating the unique features and advantages of the proposed approach. Future studies can delve into a meticulous examination of the methodologies employed in similar works, such as the references provided, to draw clearer distinctions and identify potential synergies. Additionally, incorporating advanced techniques like R-CNN into the comparative analysis can offer insights into their complementary strengths and weaknesses. Moreover, exploring variations in network architectures and training strategies could uncover opportunities for further optimization. The quest for a more refined and comprehensive understanding of heart image segmentation demands continuous exploration, and future endeavors will focus on refining the proposed approach in tandem with emerging methodologies, ultimately advancing the state of the art in this critical domain.

The deployment of U-net for encoding and decoding in the results section is grounded in its well-established effectiveness in medical image segmentation tasks. Recent research, such as the work presented in Yousef et al. [51], has further substantiated the prowess of U-net in precisely delineating anatomical structures. The architectural characteristics of U-net, including its expansive receptive field and skip connections facilitating feature concatenation, align seamlessly with the intricacies of medical images, enhancing contextual understanding. Additionally, the cited research demonstrates the adaptability of U-net across various medical imaging modalities, reinforcing its versatility. However, acknowledging the perpetual evolution of deep learning architectures, future investigations may explore and integrate the latest advancements, such as Transformer-based models, which have demonstrated promising results in diverse computer vision tasks. This nuanced discussion aims to underscore the robustness of the chosen methodologies while embracing the dynamism inherent in the ever-evolving landscape of medical image analysis.

In Table 1, we chose to specifically highlight the outcomes achieved by bicubic interpolation and LapSRN to illustrate the performance improvement facilitated by our proposed method. However, we acknowledge the importance of a more comprehensive comparison with other state-of-the-art methods. In future work, we will incorporate additional key indicators and experimental results to substantiate this claim. These may include the following: (1) Computational time comparison: We will provide quantitative measures of the time required for image reconstruction using bicubic interpolation and LapSRN, offering readers a more precise understanding of the computational efficiency of our proposed method. (2) Storage space analysis: A detailed analysis of the storage space requirements for the reconstructed images using both methods will be presented. This will encompass considerations of file size, memory usage, and any relevant metrics to emphasize the practical advantages of our approach. By incorporating these additional indicators, we aim to enhance the transparency and comprehensibility of our findings regarding the efficiency of bicubic interpolation in comparison to the LapSRN method.

While our proposed heart image segmentation method has shown promising results on the EchoNet-Dynamic and ACDC datasets, we acknowledge certain limitations associated with the dataset choices that warrant careful consideration.

(1) Limited diversity: The selected datasets, although widely used in heart image segmentation studies, might not fully capture the diversity present in real-world clinical scenarios. The EchoNet-Dynamic dataset primarily consists of echocardiographic videos, while the ACDC dataset comprises MRI data. The limited diversity in imaging modalities and patient demographics may restrict the generalizability of our model to a broader range of heart imaging conditions.
(2) Specific segmentation targets: The datasets focus predominantly on left ventricular segmentation. While this aligns with the objectives of our study, it also implies a restricted scope. Future work should explore the extension of our method to encompass a more comprehensive set of segmentation targets, such as the right ventricle and myocardium, to address the broader spectrum of heart imaging challenges.
(3) Annotation variability: Despite meticulous manual annotations by expert sonographers and cardiologists, inter-observer variability in annotations might introduce inconsistencies. Future validation on datasets with multiple annotations per image and different annotators would help assess the robustness of our method to annotation variations.

To address these limitations and fortify the credibility of our proposed segmentation method, we recognize the need for extensive validation on a broader range of datasets. Future efforts will focus on the following:

(1) Diverse datasets: inclusion of datasets representing diverse imaging modalities, acquisition protocols, and patient populations to enhance the model’s adaptability to a wider spectrum of heart imaging scenarios.
(2) Clinical data integration: collaboration with multiple medical institutions to access a more extensive and varied collection of clinical datasets, ensuring the applicability of our method across different healthcare settings.
(3) Cross-domain validation: validation on datasets from different domains, including different pathologies or disease stages, to evaluate the versatility and robustness of our method beyond the specific conditions represented in the current datasets.

In summary, while the current datasets provide a foundation for evaluating our method, we recognize the importance of expanding our validation efforts to ensure the broad applicability and reliability of our proposed heart image segmentation approach.

## 5. Conclusions

In this paper, a novel pyramid-based cardiac image segmentation algorithm is introduced, incorporating a position-aware attention mechanism. The key innovations of this method can be summarized as follows: (1) Pyramid structure: The proposed approach enhances the baseline architecture by incorporating a pyramid structure inspired by super-resolution reconstruction. Through bicubic interpolation, it generates multi-channel, multi-resolution medical images. This strategy enriches the network’s access to contextual information, consequently elevating the precision of segmentation. (2) PABlock: The introduction of the PABlock is a significant innovation. This module integrates a position-aware attention mechanism, which effectively learns position information in both the horizontal and vertical directions. It embeds position encoding, intensifying the model’s emphasis on positional details during segmentation. This results in improved segmentation accuracy. (3) Experimental validation: The experimental results presented in the paper showcase the efficacy of the proposed method. It demonstrates superior segmentation precision on 2D cardiac images derived from the EchoNet-Dynamic dataset, as well as on the ACDC dataset focusing on the left ventricle of the heart. These results highlight the method’s competitive edge and its ability to outperform other techniques. Overall, this paper contributes a novel approach to cardiac image segmentation, combining the concepts of pyramid structures and position-aware attention mechanisms. The experimental outcomes underscore the method’s potential for achieving higher segmentation accuracy, making it a compelling and advanced solution in the field.

## Figures and Tables

**Table 1:** The results related to the scaling factors of the reverse pyramid and image reconstruction methods.

Item	Method	Dice (%)	Jc (%)	Precision (%)	Recall (%)	F-Measure (%)
Baseline	-	87.93	78.75	81.06	88.61	84.67
[1x,0.5x,0.25x]	Bicubic interpolation	88.02	80.13	88.74	89.37	89.05
[1x,2x,4x]	LapSRN	90.60	86.24	90.63	89.49	90.06
[1x,2x,4x]	Bicubic interpolation	**91.45**	**88.47**	**90.79**	**92.78**	**91.77**

**Table 2:** The results of the ablation experiments for the reverse pyramid structure and PABlock.

Item	Dice (%)	Jc (%)	Precision (%)	Recall (%)	F-Measure (%)
Baseline	87.93	78.75	81.06	88.61	84.67
+ Inverted pyramid structure	90.46	83.05	88.74	**93.22**	90.92
+ PABlock	89.58	81.56	90.63	89.49	90.06
Our method	**91.45**	**88.47**	90.79	**92.78**	**91.77**

**Table 3:** The comparative results of our method on the EchoNet-Dynamic dataset against other existing approaches.

Item	Dice (%)	Jc (%)	Precision (%)	Recall (%)	F-Measure (%)
Deeplab v3	87.93	78.75	81.06	88.61	84.67
PSPNet [14]	88.19	80.13	88.91	88.19	88.55
OCNet [41]	90.23	82.48	90.26	91.05	90.65
DUNet [42]	90.77	83.52	90.47	92.00	91.23
DANet [43]	90.38	82.20	**91.57**	89.48	90.51
HANet [44]	88.81	80.93	91.34	88.10	89.69
CABlock [22]	88.71	80.22	89.63	88.95	89.29
GraphECV [45]	91.13	-	-	-	-
ViT-FRD [46]	78.52	-	-	-	-
DeepPhase [47]	-	-	76	78	-
Our method	**91.45**	**88.47**	90.79	**92.78**	**91.77**

**Table 4:** The comparative results of our method on the ACDC dataset against other existing approaches.

Item	Dice (%)	Jc (%)	Precision (%)	Recall (%)	F-Measure (%)
Deeplab v3	62.86	55.03	56.73	77.99	65.68
PSPNet [14]	52.13	42.66	56.55	54.78	55.65
OCNet [41]	53.31	44.23	57.80	56.08	56.93
DUNet [42]	53.13	44.20	58.06	55.51	56.76
DANet [43]	56.92	47.75	**60.90**	57.30	59.05
HANet [44]	40.54	30.66	49.39	40.67	44.61
CABlock [22]	44.28	35.91	49.07	49.14	49.10
DCT [48]	56.90	45.67	-	-	-
MC-Net [49]	62.85	52.29	-	-	-
SS-Net [50]	65.82	55.38	-	-	-
Our method	**66.90**	**57.21**	59.34	**82.01**	**68.86**

## Data Availability

All the datasets used in this manuscript are publicly available datasets, already in the public domain. EchoNet-Dynamic dataset was accessed on 6 January 2023: https://echonet.github.io/dynamic/. ACDC dataset was accessed on 8 January 2023: https://www.creatis.insa-lyon.fr/Challenge/acdc/databases.html.
